# Androgen-Responsive Oncogenic lncRNA RP11-1023L17.1 Enhances c-Myc Protein Stability in Prostate Cancer

**DOI:** 10.3390/ijms232012219

**Published:** 2022-10-13

**Authors:** Wenhua Huang, Qin Chen, Yali Lu, Zhe Kong, Xuechao Wan, Yan Huang, Minyan Qiu, Yao Li

**Affiliations:** State Key Laboratory of Genetic Engineering, Shanghai Engineering Research Center of Industrial Microorganisms, School of Life Science, Fudan University, Shanghai 200433, China

**Keywords:** prostate cancer (PCa), androgen-responsive lncRNA, RP11-1023L17.1, c-Myc

## Abstract

Long noncoding RNAs (lncRNAs) have been found as novel participants in the pathophysiology of prostate cancer (PCa), which is predominantly regulated by androgen and its receptor. The biological function of androgen-responsive lncRNAs remains poorly understood. Here, we identified that lncRNA RP11-1023L17.1, which is highly expressed in PCa. RP11-1023L17.1 expression, can be directly repressed by the androgen receptor in PCa cells. RP11-1023L17.1 depletion inhibited the proliferation, migration, and cell cycle progression, and promoted the apoptosis of PCa cells, indicating that RP11-1023L17.1 acts as an oncogene in PCa cells. Microarray results revealed that RP11-1023L17.1 depletion downregulated the c-Myc transcription signature in PCa cells. RP11-1023L17.1 depletion-induced cellular phenotypes can be overcome by ectopically overexpressed c-Myc. Mechanistically, RP11-1023L17.1 represses FBXO32 mRNA expression, thereby enhancing c-Myc protein stability by blocking FBXO32-mediated c-Myc degradation. Our findings reveal the previously unrecognized roles of RP11-1023L17.1 in c-Myc-dependent PCa tumorigenesis.

## 1. Introduction

Prostate cancer (PCa) is the second most frequently occurring cancer and, in recent years, the fifth leading cause of cancer deaths in males worldwide [1]. Androgen receptors (AR) and AR signaling pathways play a decisive role in the initiation and progression of PCa [2]. AR, as a transcription factor, will translocate to the nucleus once the active androgen dihydrotestosterone binds to it. It controls a number of genes involved in the regulation of proliferation and differentiation by binding to androgen response elements (ARE) on DNA. Androgen suppression via androgen deprivation therapy and AR signaling inhibition is the cornerstone of PCa treatment [3]. However, advanced PCa, despite androgen suppression, is frequently becoming castration resistant and is considered incurable [4].

Long noncoding RNAs (lncRNAs) have been linked to a wide range of physiological and pathological processes, several of which may play a role in tumor initiation and progression. Specific lncRNAs are engaged in gene regulation at various levels, ranging from epigenetic gene silencing and transcriptional regulation to post-transcriptional protein stability control [5]. Several critical lncRNAs have been identified as novel participants in PCa, which is predominantly regulated by androgen and the AR signaling pathway [6]. However, a limited number of androgen-responsive lncRNAs, such as PCAT1 and HOTAIR, have been thoroughly studied, highlighting the need for further investigation [7,8,9].

RP11-1023L17.1, located on chromosome 5p13.2, has a 5001 NT long transcript and its role in human diseases has been poorly studied. Our previous screening indicated that RP11-1023L17.1 is an androgen responsive lncRNA in PCa [10]. Here, we investigated the function and mechanism of lncRNA RP11-1023L17.1 in PCa.

## 2. Results

### 2.1. Androgen Responsive lncRNA RP11-1023L17.1 Is a Direct Target of AR

RP11-1023L17.1 is located on the short arm of chromosome 5 at position 5p13.2. In order to investigate the role of RP11-1023L17.1 in prostate tumorigenesis, we first characterized RP11-1023L17.1 in PCa LNCaP cells. The full-length transcript of RP11-1023L17.1, which is approximately 5 kilobases, was defined using Northern blot analysis (Figure S1A).

**Figure 1:**
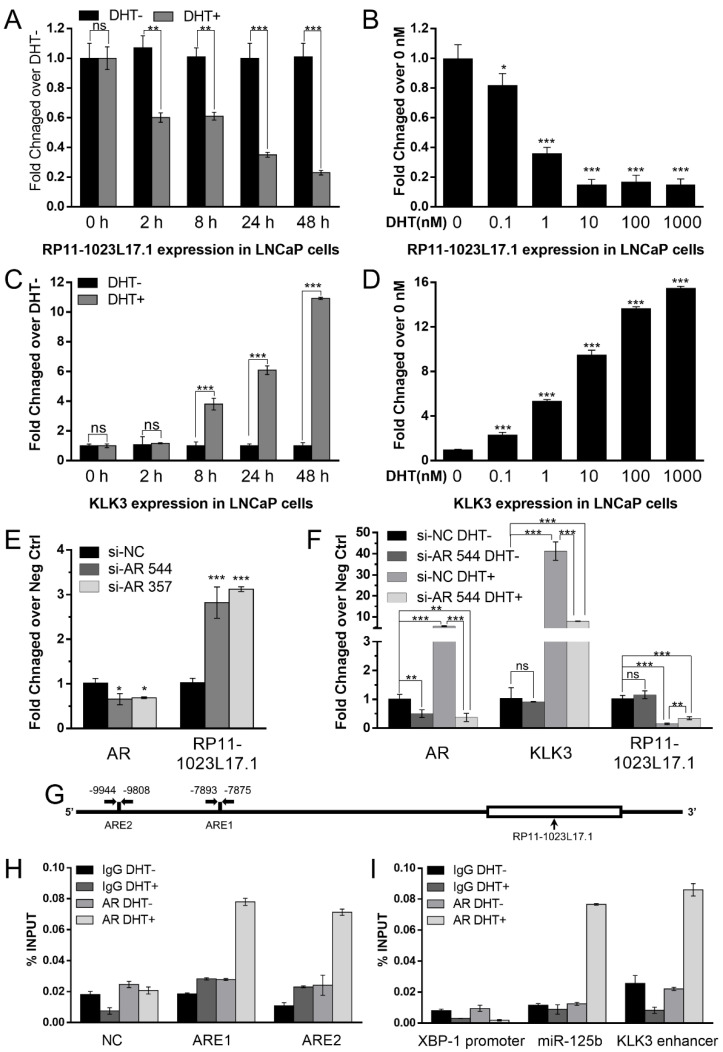
Androgen responsive lncRNA RP11-1023L17.1 is a direct AR target. (**A**–**D**) LNCaP cells were treated with 10 nM DHT (DHT+) or ethanol (DHT−) for 0, 2, 8, 24, and 48 h, or with 0, 0.1, 1, 10, 100, and 1000 nM DHT for 24 h. Expression levels of lncRNA RP11-1023L17.1 (**A**,**B**) and KLK3 (positive control, (**C**,**D**)) were evaluated by RT-qPCR. (**E**,**F**) The expression levels of AR in LNCaP cells were knocked down using small interfering RNAs si-AR 544 and si-AR 357. RT-qPCR was used to measure the expression changes of AR and RP11-1023L17.1 under normal (**E**) or androgen deprivation (**F**) conditions. KLK3 was used as a positive control. (**G**) Illustration of the potential ARE sites (ARE1: 7875–7893 bp upstream and ARE2: 9808–9944 bp upstream) located within the 10-kb region upstream of the RP11-1023L17.1 transcription start site (TSS). (**H**,**I**) ChIP-PCR analysis of LNCaP cells treated with 100 nM DHT (DHT+) or ethanol (DHT−). Cell lysates were immunoprecipitated with control IgG or AR antibodies. The presence and abundance of AREs in the RP11-1023L17.1, NC (random sequence negative control), XBP-1 (negative control), miR-125b (positive control) promoters, and KLK3 enhancer (positive control) were determined using qPCR with specific primers. Data are presented as mean ± SD (*n* ≥ 3); ns *p* > 0.05, * *p* < 0.05, ** *p* < 0.001, and *** *p* < 0.0001.

In order to explore the regulatory relationships between RP11-1023L17.1 and AR in PCa, we tested the responses of RP11-1023L17.1 on dihydrotestosterone (DHT), the potent agonist of the AR, in LNCaP cells. RP11-1023L17.1 expression was decreased by DHT treatment in a dose- and time-dependent manner (Figure 1A–D). We knocked down AR expression by siRNAs in LNCaP cells and observed that RP11-1023L17.1 expression was markedly decreased (Figure 1E). Furthermore, androgen deprivation eliminated the effect of AR knockdown on RP11-1023L17.1 expression (Figure 1F), where AR was not activated in either the NC or KD groups in the absence of DHT. These results indicated that RP11-1023L17.1 expression was repressed by androgen.

Then, we predicted potential ARE sites within the RP11-1023L17.1 promoter (10 kb region upstream of the RP11-1023L17.1 transcription start site) by employing Genomatix software, and two AREs were predicted (ARE1: 7875–7893 bp, ARE2: 9808–9944 bp) (Figure 1G). Chromatin immunoprecipitation-polymerase chain reaction (ChIP-PCR) demonstrated that AR bound directly to the predicted AREs (Figure 1H,I), indicating that RP11-1023L17.1 is a direct AR target.

### 2.2. RP11-1023L17.1 Is Highly Expressed in PCa, and Its Expression Is Regulated by p38-MAPK Signaling Pathway

**Figure 2:**
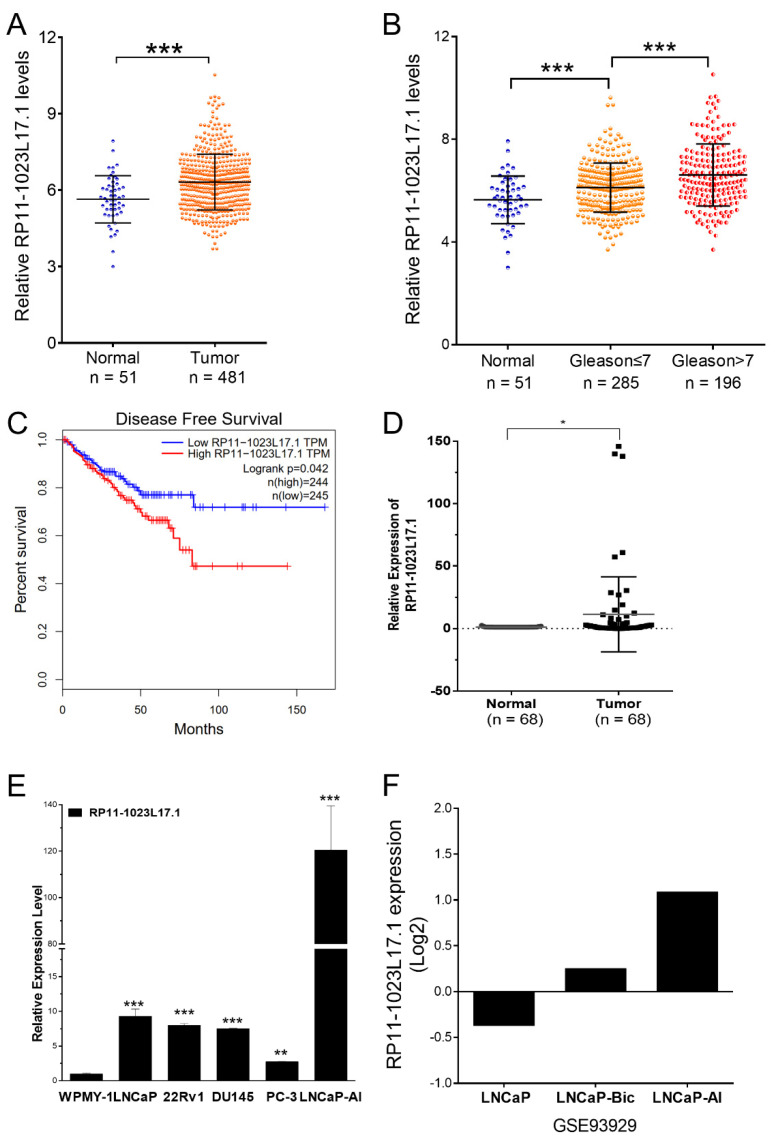
RP11-1023L17.1 is highly expressed in PCa. (**A**) Analysis of the expression levels of RP11-1023L17.1 in PCa tissues and normal tissues acquired from the TCGA database. (**B**) Analysis of the relationship between the expression of RP11-1023L17.1 and the pathological grade of PCa. (**C**) Kaplan–Meier curves for disease-free survival of PCa patients in a published dataset acquired from TCGA, using the high quartile RP11-1023L17.1 level as the cutoff. (**D**) The expression levels of RP11-1023L17.1 in 68 paired PCa tissue samples measured by RT-qPCR. (**E**) The relative expressions of RP11-1023L17.1 in normal prostate stromal cell line WPMY-1 and PCa cell lines LNCaP, 22Rv1, DU145, PC-3, and LNCaP-AI were measured by RT-qPCR. The data were normalized against WPMY-1, and β-Actin were used as housekeeping references. (**F**) The GEO dataset GSE93929 analysis showed the expression of RP11-1023L17.1 in androgen dependent (androgen sensitive) cell line LNCaP and androgen independent (castration resistant) cell lines LNCaP-Bic and LNCaP-AI. Data are presented as mean ± SD (*n* ≥ 3). * *p* < 0.05, ** *p* < 0.001, and *** *p* < 0.0001.

Comparing the expression level of RP11-1023L17.1 in 51 normal prostate tissues and 481 tumor tissues from The Cancer Genome Atlas (TCGA) dataset, we observed that RP11-1023L17.1 was highly expressed in PCa tissues compared with normal prostate tissues, and its expression was also positively correlated with the pathological grade (Figure 2A,B). Kaplan–Meier analysis indicated that a high level of RP11-1023L17 expression was significantly correlated with disease-free survival in TCGA PCa cohort. (Figure 2C). Moreover, we collected 68 paired prostate cancer tissues from patients, and higher levels of RP11-1023L17.1 were detected by RT-qPCR in prostate carcinomas compared to the matched normal prostate tissues adjacent to tumors (Figure 2D). We also analyzed publicly accessible GEO data GSE179321, which revealed the higher expression of RP11-1023L17.1 in PCa tissues compared to adjacent normal tissues (Figure S1B).

We compared the relative expression of RP11-1023L17.1 in human prostate stromal cells (WPMY-1) as well as PCa AR-dependent (LNCaP, 22Rv1) and AR-independent (DU145, PC-3, and LNCaP-AI) cells. The results showed that the expression level of RP11-1023L17.1 in PCa cells was higher than that of normal prostate cells. Moreover, RP11-1023L17.1 expression was remarkably upregulated in LNCaP-AI, an androgen-independent cell line established in our lab [11], compared with parental LNCaP cells (Figure 2E). The analysis of GEO data GSE93929 also suggested that RP11-1023L17.1 expression was upregulated in androgen-independent (castration resistant) cell lines LNCaP-Bic and LNCaP-AI, compared with the androgen-dependent (androgen sensitive) cell line LNCaP (Figure 2F).

Several signaling pathways, including Wnt, TGF-β, PI3K/Akt, mTOR, and p38-MAPK pathways, have been reported to contribute to the emergence and growth of androgen-independent PCa (AIPC) with the crosstalk of AR pathways [12,13]. In order to explore the upstream signaling pathways that regulate RP11-1023L17.1 in AIPC, we evaluated the effect of signaling pathway inhibitors on RP11-1023L17.1 expression. The results showed that the p38 inhibitor SB203580 reduced the expression of RP11-1023L17.1 in a time- and dose-dependent manner, indicating that the p38-MAPK pathway can regulate the expression of RP11-1023L17.1 in AIPC (Figure S1C–G).

### 2.3. RP11-1023L17.1 Promotes Proliferation, Migration, and EMT of PCa Cells

**Figure 3:**
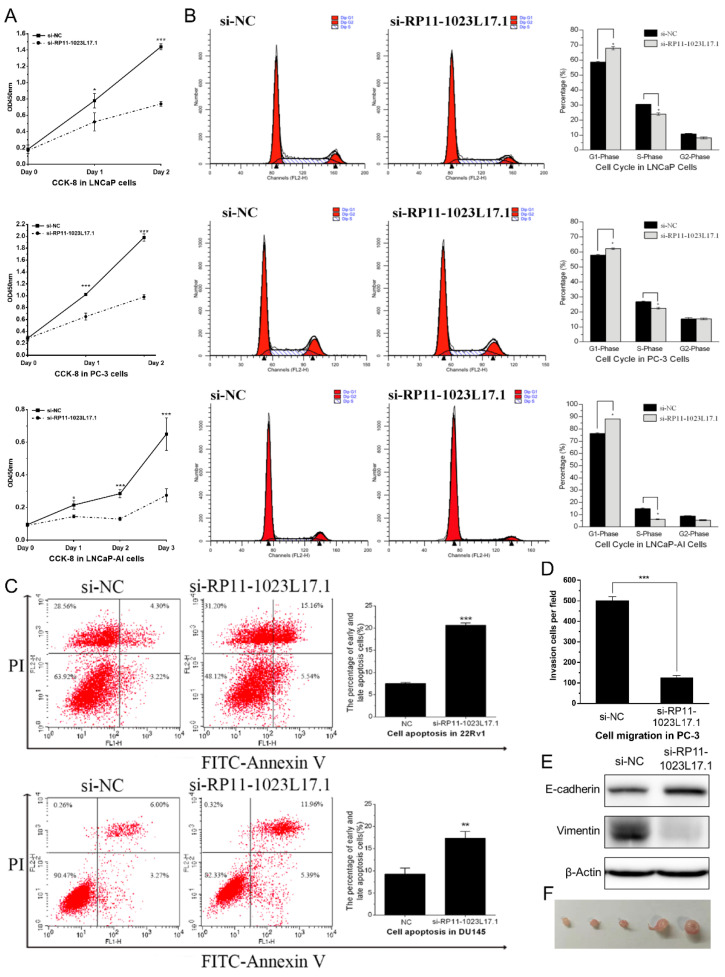
RP11-1023L17.1 promotes proliferation migration, and EMT of PCa cells. (**A**) Cell proliferation was measured by CCK-8 assay of LNCaP, PC-3, and LNCaP-AI cells transfected with si-NC or siRNA targeting RP11-1023L17.1 (si-RP11-1023L17.1). (**B**) The LNCaP, PC-3, and LNCaP-AI cells transfected with si-NC or si-RP11-1023L17.1 were stained with propidium iodide (PI), and the cell cycle distributions were evaluated by flow cytometry. (**C**) The 22Rv1 and DU145 cells transfected with si-NC or siRNA targeting RP11-1023L17.1 were stained with annexin V and PI, and the cell apoptosis was evaluated by flow cytometry. (**D**) Cell migration was monitored by transwell assay in PC-3 cells transfected with si-NC or si-RP11-1023L17.1. (**E**) Assessment of E-cadherin and vimentin expression by Western blotting in PC-3 cells transfected with si-NC or si-RP11-1023L17.1. β-actin was used as a loading control. (**F**) Tumor derived from PC-3 sh-NC cells formation in nude mice after injection. * *p* < 0.05, ** *p* < 0.001, and *** *p* < 0.0001. Data are presented as mean ± SD (*n* ≥ 3).

In order to explore the functional roles of RP11-1023L17.1 in PCa, we efficiently knocked down the expression of RP11-1023L17.1 using siRNAs (Figure S2A). Knockdown of RP11-1023L17.1 markedly inhibited the growth rate of PCa cells (Figure 3A). RP11-1023L17.1 knockdown also induced G1/S cell cycle arrest (Figure 3B and Figure S2B), increased the rates of apoptotic cells (Figure 3C and Figure S2C), and reduced cell migration (Figure 3D and Figure S2C). Epithelial to mesenchymal transition (EMT) promotes the aggressive behavior of cancer cells and can be characterized by E-cadherin and vimentin, which are an epithelial marker and a mesenchymal marker, respectively. The upregulation of E-cadherin and downregulation of vimentin by RP11-1023L17.1 knockdown revealed that RP11-1023L17.1 may affect PCa metastasis by promoting EMT (Figure 3E).

Then, we established the RP11-1023L17.1 stable knockdown PC-3 cell line (PC-3-sh-RP11-1023L17.1), and observed that its growth rate was markedly reduced compared with PC-3-sh-NC cells (Figure S2E). The PC-3-sh-RP11-1023L17.1 cells lost their ability to form tumors in nude mice after injection, whereas PC-3-sh-NC formed tumors (Figure 3F).

Altogether, these results suggest that RP11-1023L17.1 plays a proto-oncogenic role in PCa cells.

### 2.4. RP11-1023L17.1 Does Not Function as a miRNA Sponge through ceRNA Mechanism

We then explored the molecular mechanisms underlying RP11-1023L17.1-induced PCa tumorigenesis. The subcellular localization of RP11-1023L17.1 in androgen-stimulated conditions was detected by quantitative real-time PCR (RT-qPCR) following nuclear/cytoplasmic fractionation assays (Figure S3A,B). The results that DHT+ has significantly reduced RP11-1023L17.1 in the nucleus compared with DHT−, which may also be due to the decrease of newly transcribed RP11-1023L17.1, indirectly proved that DHT inhibits RP11-1023L17.1 transcription.

LncRNAs can function as competitive endogenous RNAs (ceRNA) during tumorigenesis and development [14]. If RP11-1023L17.1 can sponge miRNA, it will inhibit the activity of miRNA and positively regulate the expression of miRNA target genes; meanwhile, it might be downregulated by the miRNA. In order to investigate whether RP11-1023L17.1 plays a role in PCa through the ceRNA mechanism, we identified candidate miRNAs that may target RP11-1023L17.1 by miRDB [15] (Table S3). Furthermore, we analyzed the expressions of these miRNAs and the correlation coefficients with RP11-1023L17.1 using the TCGA dataset. The results showed that the expressions of miR-205-5p, miR-27b-3p, and miR-944 significantly decreased in PCa tissues and were weakly negatively correlated with RP11-1023L17.1 (r = −0.1349, −0.2153, −0.1042, respectively). We then collected experimentally verified miRNA target genes with at least two pieces of strong evidence by miRTarBase [16]. The correlation coefficients of these target genes and RP11-1023L17.1, most of which were weakly correlated, are listed in Table S4. We also analyzed the expressions of these target genes in RP11-1023L17.1 knockdown microarray, as described below (Table S4). The expression profile data showed that these target mRNAs did not show a down- or upward trend in general (Figure S3C).

Furthermore, we constructed fragments (P-1, P-2, P-3, and P-4) of RP11-1023L17.1 as miRNA sponges to competitively bind miRNA with RP11-1023L17.1 in LNCaP and PC-3 cells (Figure S4A–E). The RT-qPCR results showed that high expressions of the fragments failed to affect the expression of RP11-1023L17.1 and the target mRNAs. We then knocked down the RNase type 3 enzyme Dicer, which did not upregulate the expression of RP11-1023L17.1, but partially inhibited RP11-1023L17.1 expression (Figure S4F,G). In addition, Dicer knockdown and simultaneous overexpression of RP11-1023L17.1 fragments did not promote the expression of RP11-1023L17.1 (Figure S4H,I).

Altogether, these results indicate that RP11-1023L17.1 may not function mainly as a miRNA sponge through the ceRNA mechanism.

### 2.5. Identificaton of Genes Regulated by RP11-1023L17.1

We performed the transcriptomic analysis by microarray in order to identify downstream genes which are regulated by RP11-1023L17.1 in LNCaP cells. The results showed 5171 transcripts with significant changes (>2-fold) in expression compared with the controls, 1153 genes showed a >3-fold increase, and 650 genes exhibited a >3-fold decrease in RP11-1023L17.1 knockdown cells compared with control cells (Figure S5A, Table S5). We randomly selected eight genes and validated the reliability of microarray data by RT-qPCR in LNCaP and LNCaP-AI cells (Figure S5B,C). Gene Ontology (GO) indicated that the differential genes affected by RP11-1023L17.1 knockdown were mainly enriched in cell cycle progression (Figure S5D), which was consistent with our results that RP11-1023L17.1 promoted cell cycle progression in PCa. The Kyoto Encyclopedia of Genes and Genomes (KEGG) pathway enrichment analysis revealed that the differential genes affected by RP11-1023L17.1 knockdown were significantly enriched in the TNF-α/NF-κB, AR, and WNT pathways (Figure S5E).

### 2.6. Post-Translational Regulation of c-Myc Protein Stability by RP11-1023L17.1 through Proteasomal Degradation

We integrated the expression profiling data with Genomatix software in order to identify transcription factors that may be affected by RP11-1023L17.1 in PCa cells. As a result, 153 transcription factors were obtained and ranked based on the number of target genes whose expression changed significantly. The 53 transcription factors with more than 10 regulated genes, whose expression changed significantly by RP11-1023L17.1 knockdown, are listed in Supplementary Table S6. The top 15 transcription factors are shown in Table 1, and MYC ranked first in the list.

The MYC gene family serves as an important regulator of tumorigenesis and includes three members, namely, c-Myc, N-Myc, and L-Myc, of which c-Myc is highly expressed in a variety of cancers and functions as a proto-oncogene [17]. Multiple lncRNAs have been reported to interact with c-Myc and play important roles in diverse pro-tumorigenic processes [18,19]. The RP11-1023L17.1 knockdown microarray analysis showed that RP11-1023L17.1 affected the expressions of numerous c-Myc transcriptional targets, and this result was further validated by RT-qPCR (Figure S6A–C). By calculating Pearson’s correlation coefficients for genes co-expressed in PCa in TCGA, we observed that RP11-1023L17.1 was positively correlated with the expression of most c-Myc target genes (Figure S6D).

**Figure 4:**
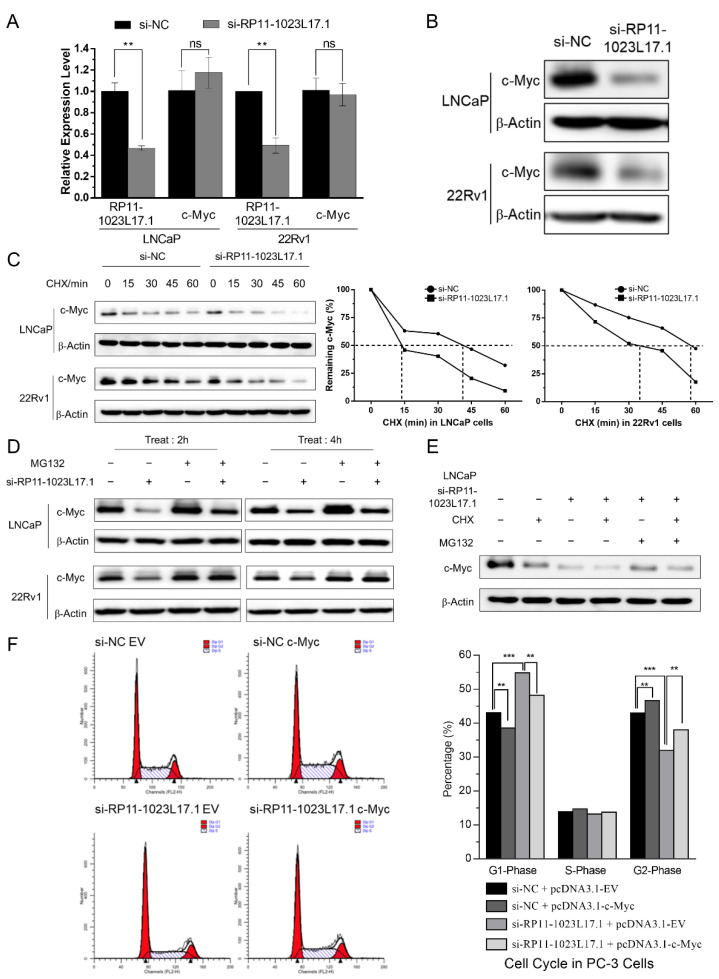
The enhancement of RP11-1023L17.1 on c-Myc protein stability may depend on proteasomal degradation. (**A**) The relative expression of RP11-1023L17.1 and c-Myc in LNCaP and 22Rv1 cells transfected with si-NC or si-RP11-1023L17.1 was measured by RT-qPCR. Data are presented as mean ± SD (*n* ≥ 3). (**B**) Assessment of c-Myc expression by Western blotting in LNCaP and 22Rv1 cells transfected with si-NC or si-RP11-1023L17.1. β-actin was used as a loading control. (**C**) Assessment of c-Myc expression by Western blotting in LNCaP and 22Rv1 cells transfected with si-NC or si-RP11-1023L17.1, following treatment with 25 µg/mL protein synthesis inhibitor cycloheximide (CHX) in a time series. (**D**) Assessment of c-Myc expression by Western blotting in LNCaP and 22Rv1 cells transfected with si-NC or si-RP11-1023L17.1, following treatment with DMSO or 10 µM proteasome inhibitor MG132 for 2 h or 4 h. (**E**) Assessment of c-Myc expression by Western blotting in LNCaP cells transfected with si-NC or si-RP11-1023L17.1 following treatment with DMSO, 25 µg/mL CHX and/or 10 µM MG132 for 0.5 h. β-Actin was used as a loading control. (**F**) PC-3 cells co-transfected with EV or pcDNA3.1-MYC and si-NC or si-RP11-1023L17.1 were stained with PI, and the cell cycle distributions were evaluated by flow cytometry. ns *p* > 0.05, ** *p* < 0.001, and *** *p* < 0.0001. Data are presented as mean ± SD (*n* ≥ 3).

In order to examine the effect of RP11-1023L17.1 on c-Myc, we knocked down RP11-1023L17.1 in LNCaP and 22Rv1 cells and examined changes in the mRNA and protein levels of c-Myc. The results showed that the Myc protein level was markedly reduced, whereas its mRNA level remained unchanged (Figure 4A,B), suggesting that RP11-1023L17.1 may regulate c-Myc expression at the translational or post-translational level.

In order to investigate whether RNA affects the protein stability of c-Myc, we treated cells with the protein synthesis inhibitor cycloheximide (CHX) to observe the protein half-life. RP11-1023L17.1 knockdown sharply reduced the protein half-life of c-Myc, which was shortened from 40.5 min to 13.5 min in LNCaP cells and from 57 min to 34 min in 22Rv1 cells (Figure 4C and Figure S6E,F), indicating that RP11-1023L17.1 prolongs the half-life of c-Myc protein. Furthermore, c-Myc downregulation by RP11-1023L17.1 knockdown in LNCaP and 22Rv1 cells can be reversed by treatment with the proteasome inhibitor MG132 (Figure 4D). MG132 treatment also partially restored the c-Myc protein decrease caused by the protein synthesis inhibitor CHX and RP11-1023L17.1 knockdown (Figure 4E and Figure S6G). These results illustrated the regulation of c-Myc protein stability by RP11-1023L17.1 at the post-translational level.

In order to explore whether c-Myc rescues the cellular phenotype caused by RP11-1023L17.1 knockdown, we overexpressed c-Myc in RP11-1023L17.1 knockdown cells and detected changes in cell cycle progression (Figure 4F and Figure S6H,I). The results showed that high expression of c-Myc partially restored the cell cycle inhibition caused by RP11-1023L17.1 knockdown, indicating that RP11-1023L17.1 functions at least in part through c-Myc in PCa cells.

### 2.7. RP11-1023L17.1 Inhibits c-Myc Protein Degradation through Regulation of FBXO32

**Figure 5:**
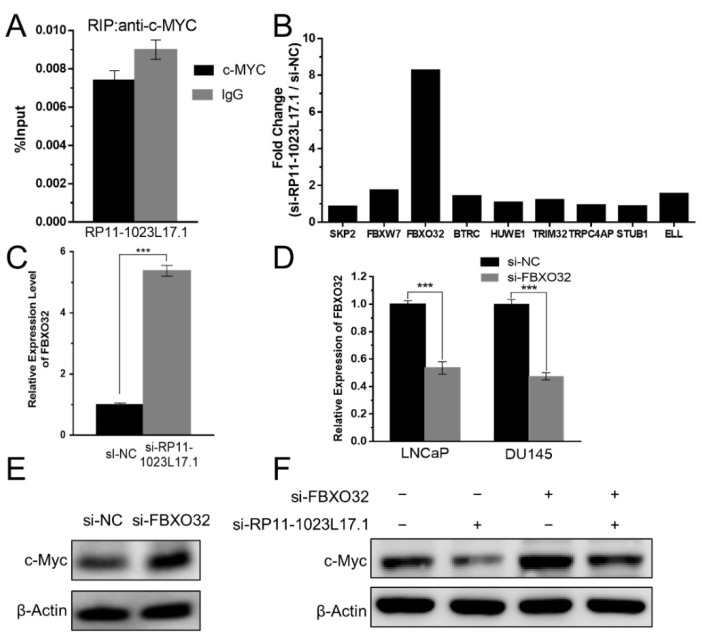
RP11-1023L17.1 inhibits c-Myc protein degradation through the regulation of FBXO32. (**A**) RP11-1023L17.1 was detected in RIP assays using anti-c-Myc or IgG antibodies in LNCaP cells. (**B**) The expression changes of ubiquitin ligase genes that regulate c-Myc stability in RP11-1023L17.1 knockdown microarray analysis. (**C**) The relative expression of FBXO32 in LNCaP cells transfected with si-NC or si-RP11-1023L17.1 was measured by RT-qPCR. (**D**) The siRNA silencing efficacy of FBXO32 in LNCaP and DU145 cells was measured by RT-qPCR. Data are presented as mean ± SD (*n* ≥ 3). *** *p* < 0.0001. (**E**) Assessment of c-Myc expression by Western blotting in LNCaP cells transfected with si-NC or si-FBXO32. (**F**) Assessment of c-Myc expression by Western blotting in LNCaP cells co-transfected with si-NC, si-FBXO32 or si-RP11-1023L17.1. β-Actin was used as a loading control.

In order to explore the mechanism by which RP11-1023L17.1 increases the stability of c-Myc protein, we tested the possible interaction between RP11-1023L17.1 and c-Myc by RNA co-immunoprecipitation (RIP) assay (Figure 5A). The results showed that c-Myc failed to enrich RP11-1023L17.1 in LNCaP cells, indicating that RP11-1023L17.1 may regulate the protein stability of c-Myc in an indirect manner.

c-Myc protein is constantly degraded through the ubiquitin–proteasome pathway. The expression of FBXO32, but not the other reported E3 ubiquitin ligases for c-Myc, significantly increased after the knockdown of RP11-1023L17.1 (Figure 5B,C). Furthermore, we knocked down FBXO32 expression and detected the protein changes in c-Myc in RP11-1023L17.1 knockdown cells (Figure 5D,F). The results showed that simultaneous knockdown of FBXO32 and RP11-1023L17.1 reverted the effect of RP11-1023L17.1 knockdown on c-Myc protein stability, indicating that RP11-1023L17.1 may inhibit c-Myc protein degradation by repressing FBXO32 expression.

## 3. Discussion

RP11-1023L17.1 was reported to be significantly upregulated during coxsackievirus B3 (CVB3) infection. It potentially affects CVB3 replication, and plays roles in CVB3-p53 mutual regulation [20]. However, its pathophysiological functions in cancer have not been invested prior to our study. LncRNAs have been found as novel participants and are predominantly regulated by androgen and its corresponding receptor AR. AR can function as both a tumor suppressor and a proliferation stimulator during PCa progression, and AR-downregulated genes can either be involved in tumor suppression or tumor progression [21]. The androgen-dependent repression of proto-oncogenes like c-Met represents a protective function of AR signaling [22]. In this study, we observed that the expression of RP11-1023L17.1 was repressed by AR, and was a direct transcriptional target of AR. RP11-1023L17.1 is highly expressed in PCa and upregulated in androgen-independent cell lines. RP11-1023L17.1 knockdown inhibited the proliferation, migration, and progression of the cell cycle, and promoted the apoptosis of PCa cells. Collectively, we proposed that androgen-repressed lncRNA RP11-1023L17.1 acts as an oncogene, and may play a major role in androgen-independent PCa cells.

In PCa, p38-MAPK activity is dysregulated and presents both oncogenic and tumor-suppressor roles [23,24,25,26]. p38-MAPK promotes Hsp27 activation and the hypoxia-mediated increase in AR activity in castration resistant PCa cells [27]. In breast cancer, lncRNA ST8SIA6-AS1 promotes cell proliferation, migration, and invasion through the p38-MAPK signaling pathway [28]. In this study, the p38-MAPK signaling pathway may be the upstream signaling pathway regulating the expression of RP11-1023L17.1 in AIPC cells, but the detailed molecular mechanisms should be explored in future studies.

The ceRNA hypothesis describes that lncRNAs, messenger RNAs, and transcribed pseudogenes compete for binding to miRNAs via miRNA response elements [29]. Several lncRNAs function as ceRNAs to facilitate or inhibit PCa progression [30,31,32,33]. In order to investigate whether RP11-1023L17.1 can function through sponge miRNAs, we hypothesized that miR-205-5p, miR-27b-3p, and miR-944 may sponge RP11-1023L17.1, but the correlation of their expression with RP11-1023L17.1 was relatively weak. Dicer is a multidomain ribonuclease III enzyme that is involved in miRNA and siRNA biogenesis [34]. However, Dicer knockdown and high expressions of RP11-1023L17.1 fragments failed to promote the expression of RP11-1023L17.1. RP11-1023L17.1 may not function through ceRNA mechanisms in PCa cells. Furthermore, RP11-1023L17.1 failed to upregulate the expression of miRNA target genes, indicating that RP11-1023L17.1 does not function as a ceRNA in PCa cells.

In addition to AR, c-Myc plays important roles in PCa progression. Early on in the course of PCa, c-Myc is overexpressed and functions as a critical driver of carcinogenesis and disease progression. Mutations of c-Myc alter the binding of transcription factors such as YY1, FoxA1, and Tcf4 to their regulated genes, which in turn alter the responses of genes to signaling pathways, such as AR and Wnt [35,36]. In addition to the conventional theories of increased AR signaling or the formation of a bypass pathway, Bernard et al. showed that c-Myc may also stimulate androgen-independent growth through a downstream mechanism. Androgen-dependent PCa cells with high expressing c-Myc grew independently of androgens, and displayed tumorigenic capabilities in androgen-depleted conditions. C-Myc is expressed in and necessary for AIPC cell growth [37]. Several lncRNAs are reported to regulate c-Myc in PCa cells. The tumor-promoting lncRNA PCGEM1 regulates cancer cell metabolism by promoting the chromatin recruitment of c-Myc and increasing its transactivation activity [38]. Cytoplasmic lncRNAs may have roles in protein stability and modification [19]. Our study revealed that 153 transcription factors were identified that may be affected by RP11-1023L17.1 in PCa cells, and MYC ranked first in the list. RP11-1023L17.1 did not affect the expression of c-MYC mRNA, but promoted c-Myc protein stability. Moreover, c-Myc regulates G1/S transition [18], and our results showed that high expression of c-Myc partially restored the cell cycle inhibition caused by RP11-1023L17.1 knockdown. That is, RP11-1023L17.1 promoted cell proliferation, at least in part, through c-Myc in PCa.

FBXO32 targets c-Myc for proteasomal degradation and inhibits c-Myc activity [39]. It acts as an E3 ubiquitin ligase and suppresses breast cancer and lung cancer tumorigenesis [40,41]. FBXO32 can be modulated by LINC00494 to facilitate ovarian cancer progression via binding with NF-κB [42,43]. Our study showed that RP11-1023L17.1 promoted c-Myc protein stability by regulating FBXO32 expression.

In conclusion, AR-regulated RP11-1023L17.1 plays an oncogenic role in PCa through c-Myc.

## 4. Materials and Methods

### 4.1. Cell Culture, Drug Treatment, and Transfection

PCa cell line LNCaP cells were obtained from the American Type Cult Collection. Normal prostate stromal cell line WPMY-1 and PCa cell lines 22RV1, DU145, and PC-3 were purchased from Cell Bank of Chinese Academy of Sciences. The androgen-independent prostatic carcinoma cell line LNCaP-AI was established in our lab. The cell lines were authenticated by short tandem repeat (STR) analysis. WPMY-1, LNCaP, 22Rv1, DU145, and PC-3 cell lines were grown in RPMI 1640 culture medium (Gibco, New York, NY, USA) and supplemented with 10% fetal bovine serum (FBS) (Hyclone, Logan, UT, USA), 0.1 mM non-essential amino acids (Gibco, New York, NY, USA), 1% penicillin-streptomycin solution (Gibco, New York, NY, USA), and 1 mM sodium pyruvate (Gibco, New York, NY, USA). LNCaP-AI and androgen-starved LNCaP cells were grown in a phenol red-free RPMI 1640 culture medium (Gibco, New York, NY, USA) and supplemented with 10% carbon adsorption fetal bovine serum (Hyclone, Logan, UT, USA). Cells were maintained in a humidified incubator at 37°C with 5% CO_2_.

For the purpose of androgen stimulation, LNCaP cells were starved for 24 h following DHT treatment, while ethanol was used as a control. For signal pathway inhibitor treatments, KY02111, Rapamycin, GW788388, LY294002, and SB20580 (MCE, Princeton, NJ, USA) were given at appropriate times and in appropriate concentrations, and DMSO was used as a control.

Cell transfection was performed according the transfection reagent protocol. Briefly, cells were transiently transfected using Lipofectamine 2000 (Invitrogen, Carlsbad, CA, USA) and incubated for 48 h before further operation. Sequences of siRNA are listed in Table S1.

### 4.2. Cell Nuclear and Cytoplasmic Extraction, Quantitative Real-Time PCR (qRT–PCR), and Western Blot Analysis

Cell nuclear and cytoplasmic fractions was prepared by the Nuclear and Cytoplasmic Protein Extraction Kit (Beyotime, Nantong, China), and an RNase inhibitor was added in order to prevent RNA degradation.

Total RNAs were isolated with TRIzol (Invitrogen, Carlsbad, CA, USA), then reverse-transcribed by a PrimeScript RT Reagent Kit (Takara, San Jose, CA, USA), and qPCR was performed using the SYBR Green PCR Master Mix (Vazyme, Nanjing, China) and gene-specific primers (Supplementary Table S2).

Protein was isolated with a RIPA lysis buffer (Beyotime, Nantong, China). For Western blot analysis, the isolated proteins were separated by SDS-PAGE and electro-transferred to polyvinyl difluoride membranes (PVDF). The following antibodies were used: anti-CDH1 (Proteintech, Chicago, IL, USA, 20648-1-AP), anti-VIM (Proteintech, Chicago, IL, USA, 10366-1-AP), anti-c-Myc (CST, Danvers, MA, USA, #9402), and anti-β-Actin (Abcam, Cambridge, UK, ab8227).

### 4.3. Flow Cytometry Analysis, Cell Migration, and Cell Proliferation Assay

For the cell cycle, cells were stained with 50 ng/mL propidium iodide (PI), and for cell apoptosis, cells were stained with the FITC-Annexin V Apoptosis Detection Kit (BD, Franklin Lakes, NJ, USA). The cells were evaluated by the FACSCalibur flow cytometer (BD, Franklin Lakes, NJ, USA).

For the cell migration assay, approximately 1 × 10^4^ cells/well were seeded in the transwell chambers and cultured for 48 h. Cells were stained with DAPI and observed with an Olympus inverted fluorescence microscope (Olympus, Tokyo, Japan).

The cell proliferation assay was performed using a Cell Counting Kit-8 (Dojindo, Kumamoto, Japan), and was measured at 450 nm with a Microplate Reader ELx808 (Biotek, Winooski, VT, USA).

### 4.4. ChIP and RIP Assay

Chromatin immunoprecipitation (ChIP) and an RNA immunoprecipitation (RIP) assay were performed as described previously [44]. The following antibodies were used: anti-AR (Abcam, ab108341), anti-c-Myc (CST, #9402), and anti-IgG (Abcam, Cambridge, UK, ab2410). Sequences of the primers used in the study are listed in Supplementary Table S2.

### 4.5. Microarray and Gene Ontology Assays

Total RNAs were isolated with TRIzol (Invitrogen, Carlsbad, CA, USA), checked by an Agilent Bioanalyzer 2100 (Agilent), and further purified by an RNeasy micro kit (Qiagen, Venlo, The Netherlands) and an RNase-Free DNase Set (Qiagen, Venlo, The Netherlands). The microarray was performed by the Shanghai Biotechnology Corporation using Agilent Whole Human Genome Microarray 4 × 44K for expression analysis. Genes with fold change ≥2 and *p* < 0.05 were identified as differentially expressed genes (DEGs). A total of 41,093 probes were used for expression profiling, in which 5171 transcripts showed significant changes (>2-fold) in expression compared with the controls, 1153 genes showed a >3-fold increase, and 650 genes exhibited a >3-fold decrease in RP11-1023L17.1 knockdown cells compared with control cells. Gene Ontology (GO) and Kyoto Encyclopedia of Genes and Genomes (KEGG) enrichment analyses of the DEGs were performed in order to better understand the biological functions of genes. The DEGs are listed in Supplementary Table S5.

### 4.6. Nude Mice Xenograft Model

BALB/c nude mice (4-week-old males) were bought from Shanghai Laboratory Animal Center. Ten mice were randomly divided into two groups. PC-3-sh-RP11-1023L17.1 or PC-3-sh-NC cells (5 × 10^6^ cells in 0.1 mL of PBS) were injected subcutaneously into the upper right shoulders of the mice. All experimental procedures were approved by the Ethics Committee of Fudan University (Shanghai, China).

### 4.7. Patients and Tissue Samples

All of the 68 paired PCa samples were collected from Fudan University Shanghai Cancer Center (Shanghai, China). Matched adjacent noncancerous tissues were obtained at a distance of 0.5 cm from the tumor margin. After surgery, the samples were immediately frozen in liquid nitrogen to stop the RNA from degrading. TRIzol was used to extract the RNA from the samples. The study was conducted in accordance with the Declaration of Helsinki, and approved by the Ethics Committee of Fudan University Shanghai Cancer Center (Shanghai, China).

### 4.8. Statistical Analysis

Data were expressed as the mean ± standard deviation (SD) from at least three independent experiments. Statistical comparisons of normalized data groups were made using the *t*-test or the Mann–Whitney U test, depending on the test condition. A *p*-value of less than 0.05 was considered statistically significant with a 95% confidence level. All statistical tests were two-sided. ns (no significance) >0.05, * *p* < 0.05, ** *p* < 0.001, and *** *p* < 0.0001.

## Figures and Tables

**Table 1:** The top 15 transcription factors ranked by the number of regulated genes, whose expression changed significantly by RP11-1023L17.1 knockdown.

Transcription Factor	The Number of Genes ^1^
MYC (V$EBOX)	169
JUN (V$CREB, V$AP1F)	89
SP1 (V$SP1F)	81
E2F1 (V$E2FF)	79
EGR1 (V$EGRF)	77
FOS (V$AP1F)	73
STAT3 (V$STAT)	56
CEBPB (V$CEBP)	55
CREB1 (V$CREB)	54
ESR1 (V$EREF)	44
STAT1 (V$IRFF, V$STAT)	41
TBP (O$VTBP)	38
IRF1 (V$IRFF)	36
SOX2 (V$SORY, V$STEM)	31
YY1 (V$YY1F)	29

^1^ Gene has a validated binding site with the transcription factor and had at least 1 piece of evidence of interaction.

## Data Availability

Data are contained within the supplementary material.

## Supplementary Materials

The following supporting information can be downloaded at: https://www.mdpi.com/article/10.3390/ijms232012219/s1.
