# Supplementary material for: Androgen-Responsive Oncogenic lncRNA RP11-1023L17.1 Enhances c-Myc Protein Stability in Prostate Cancer
Source: Int J Mol Sci. 2022 Oct 13;23(20):12219. doi: 10.3390/ijms232012219 (PMC9603324; doi:10.3390/ijms232012219)
Supplement: Supplementary file 1 [file ijms-23-12219-s001.zip › ijms-1904132-supplemantary.pdf]

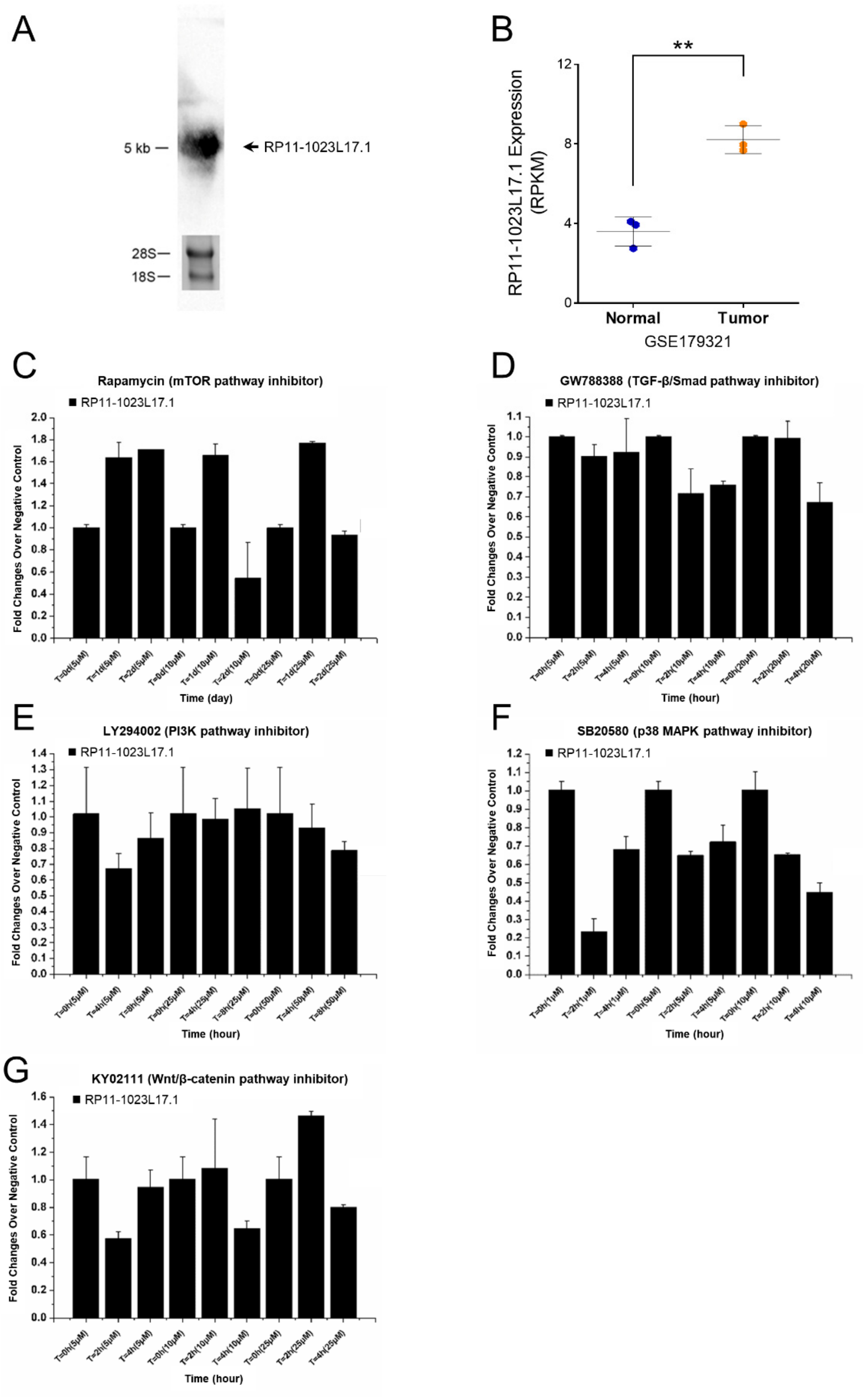

Supplementary Figure S1. p38-MAPK signaling pathway may regulate RP11-1023L17.1 expression in androgen-independent PCa. (**A**)The full-length transcript of RP11-1023L17.1 was defined in LNCaP cells using northern blot analysis. 18S and 28S ribosomal RNAs were used as internal controls. (B)The GEO dataset GSE179321 analysis showed the expression of RP11-1023L17.1 in prostate cancer tissues and adjacent normal tissues. (**B-F**) PC-3 cells were treated with pathway-specific inhibitors KY02111 (Wnt/β-catenin pathway, B), Rapamycin GW788388 (TGF-β/Smad pathway, D), LY294002 (PI3K pathway, E),

SB20580 (p38 MAPK pathway, F), or (mTOR pathway, G) in concentration and time series. The changes of RP11-1023L17.1 expression were measured by qRT-PCR. Data are presented as mean ± SD ($n \geq 3$).

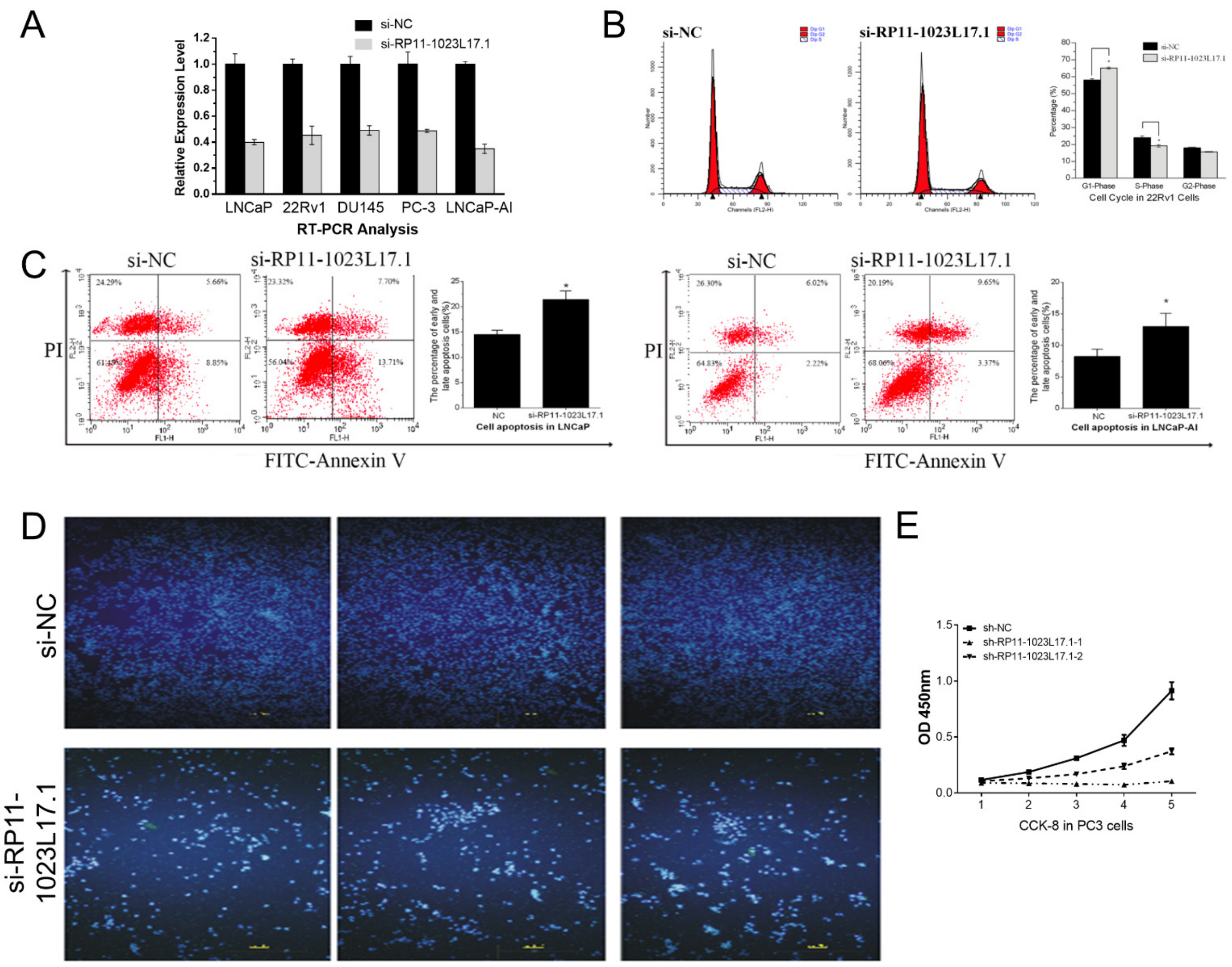

A
si-NC
si-RP11-1023L17.1
Relative Expression Level
LNCaP
22Rv1
DU145
PC-3
LNCaP-AI
RT-PCR Analysis
B
si-NC
si-RP11-1023L17.1
si-NC
si-RP11-1023L17.1
Percentage (%)
G1-Phase
S-Phase
G2-Phase
Cell Cycle in 22Rv1 Cells
C
si-NC
si-RP11-1023L17.1
PI
24.29%
5.66%
61.19%
8.85%
23.32%
7.70%
56.04%
13.71%
The percentage of early and late apoptosis cells(%)
NC
si-RP11-1023L17.1
Cell apoptosis in LNCaP
FITC-Annexin V
si-NC
si-RP11-1023L17.1
PI
26.30%
6.02%
64.83%
2.22%
20.19%
9.65%
68.06%
3.37%
The percentage of early and late apoptosis cells(%)
NC
si-RP11-1023L17.1
Cell apoptosis in LNCaP-AI
FITC-Annexin V
D
si-NC
si-RP11-1023L17.1
E
sh-NC
sh-RP11-1023L17.1-1
sh-RP11-1023L17.1-2
OD 450nm
CCK-8 in PC3 cells

Supplementary Figure S2. RP11-1023L17.1 may play a proto-oncogenic role in PCa. (**A**) The siRNA silencing efficacy of RP11-1023L17.1 in PCa cell lines was measured by qRT-PCR. (**B**) The 22Rv1 cells transfected with si-NC or si-RP11-1023L17.1 were stained with propidium iodide (PI), and the cell cycle distributions were evaluated by flow cytometry. **(C)** The LNCaP and LNCaP-AI cells transfected with si-NC or siRNA targeting RP11-1023L17.1 were stained with annexin V and PI, and the cell apoptosis was evaluated by flow cytometry. (**D**) The cell migration was monitored by transwell assay in PC-3 cells transfected with si-NC or si-RP11-1023L17.1. (**E**) The cell proliferations of PC-3 sh-NC or sh-RP11-1023L17.1 cell line were measured by CCK-8 assay. Data are presented as mean ± SD (n ≥3).

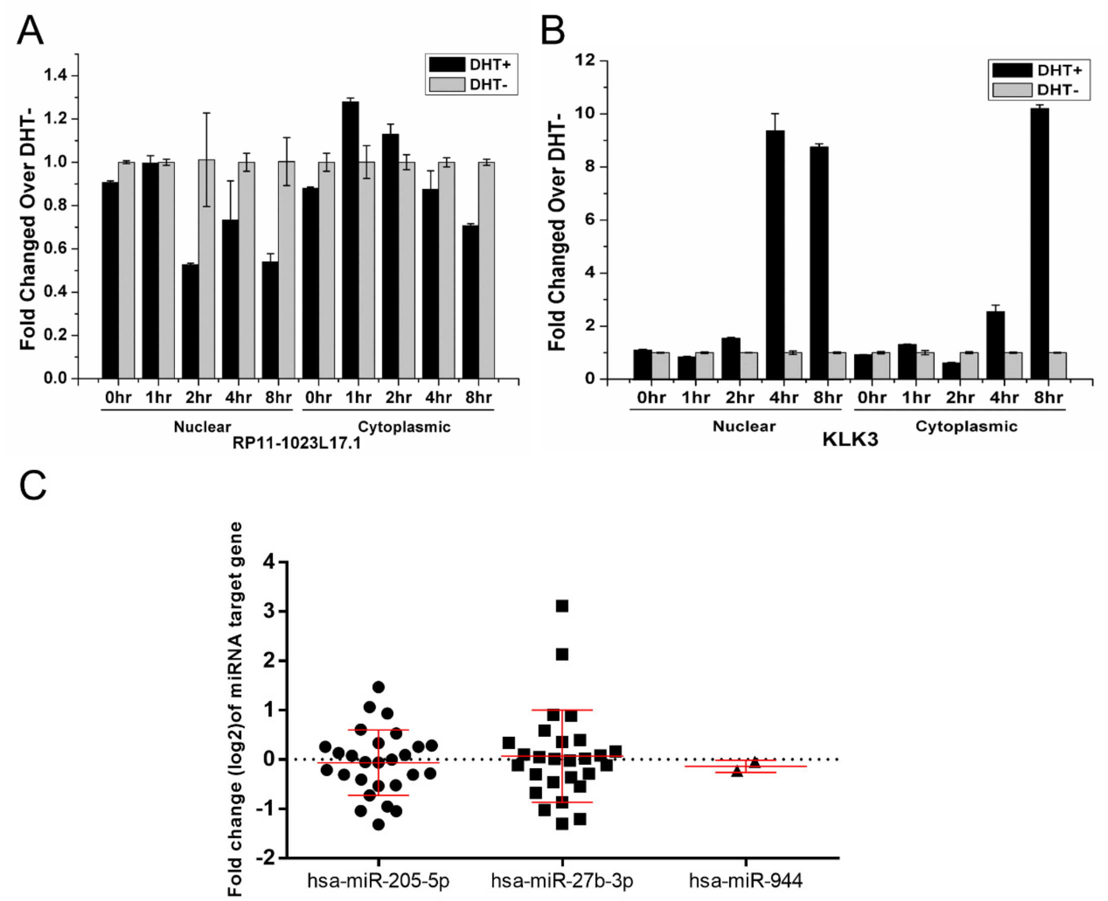

Supplementary Figure S3. RP11-1023L17.1 may not function as a miRNA sponge through the ceRNA mechanism-1. (**A-B**) LNCaP cells were treated with 10 nM DHT (DHT+) or ethanol (DHT-) for a time series, and nuclear and cytoplasmic RNA fractions were isolated. The relative expressions of RP11-1023L17.1 (A) and KLK3 (positive control, B) were measured by qRT-PCR. Data are presented as mean ± SD (n ≥3). (**C**) Fold change (log2) of miRNA target gene in RP11-1023L17.1 knockdown microarray. Data are presented as mean ± SD (n ≥3).

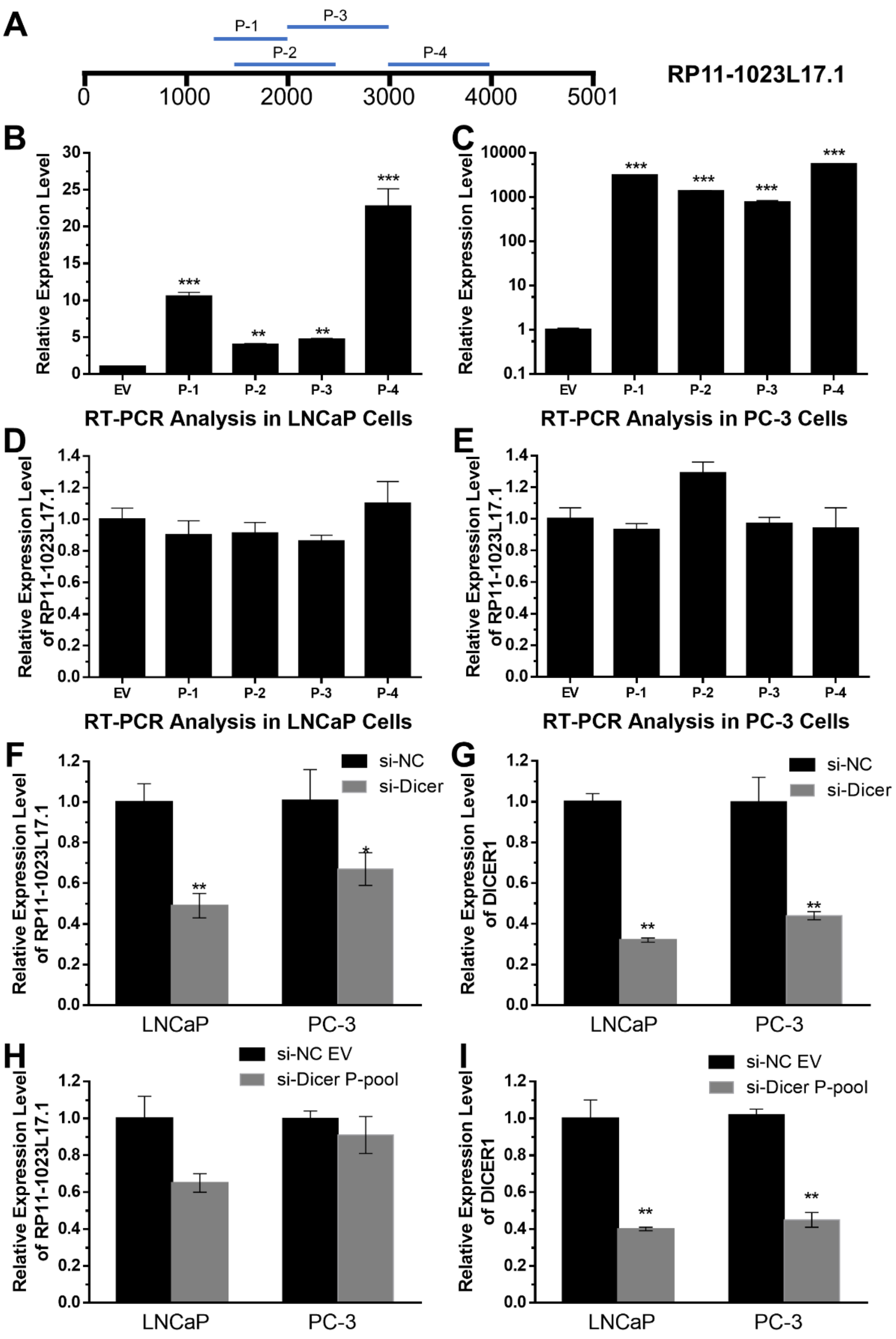

Supplementary Figure S4. RP11-1023L17.1 may not function as a miRNA sponge through the ceRNA mechanism-2. (A) Illustration of the P-1, P-2, P-3, P-4 fragments of lncRNA RP11-1023L17.1. (B-E) The expression efficacy of P-1, P-2, P-3, P-4 fragments (B, C) and the relative expression of RP11-1023L17.1 (D, E) in LNCaP (B, D) and PC-3 (C, E) cells transfected with P-1, P-2, P-3, P-4 fragments expression plasmids were measured by qRT-PCR. (F-G) The relative expression of RP11-1023L17.1 (F) and Dicer (G) in LNCaP and PC-3 cells transfected with si-NC or siRNA targeting Dicer (si- Dicer) were measured by qRT-PCR. (H-I) The relative expression of RP11-1023L17.1 (H) and Dicer (I) in LNCaP and PC-3 cells co-transfected with si-NC and empty vector (EV), or si- Dicer and pool of P-1, P-2, P-3, P-4 fragments expression plasmids were measured by qRT-PCR. Data are presented as mean ± SD (n ≥3).

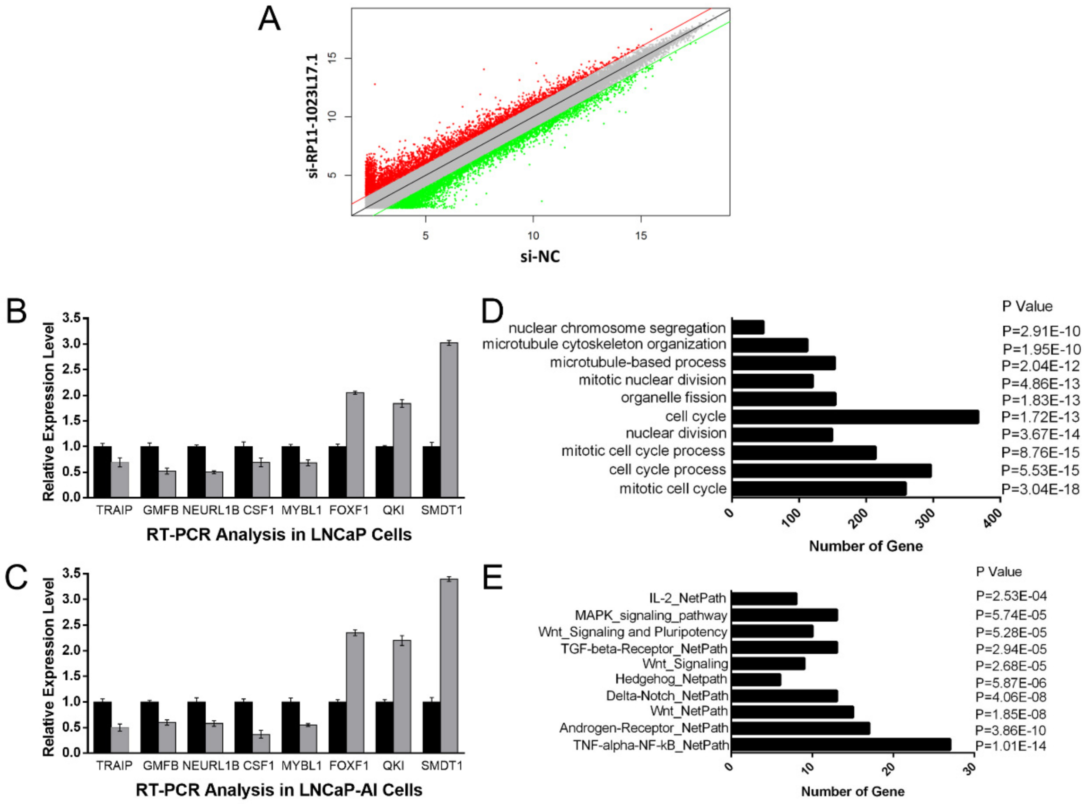

Supplementary Figure S5. Microarray analysis after RP11-1023L17.1 knockdown in LNCaP cells. (**A**) Scatter plot comparing the normalized (RPKM) expression values for all genes of RNA-seq analysis in LNCaP cells transfected with si-NC or si-RP11-1023L17.1, of which 2780 genes were upregulated (red points) and 2391 downregulated (green points) for more than 2-fold. (**B-C**) The relative expression of eight randomly selected genes in LNCaP (B) and LNCaP-AI (C) cells transfected with si-NC or si-RP11-1023L17.1 were measured by qRT-PCR. Data are presented as mean ± SD (n ≥3). (**D-E**) GO (D) and KEGG (E) pathway enrichment analysis of microarray differentially expressed genes.

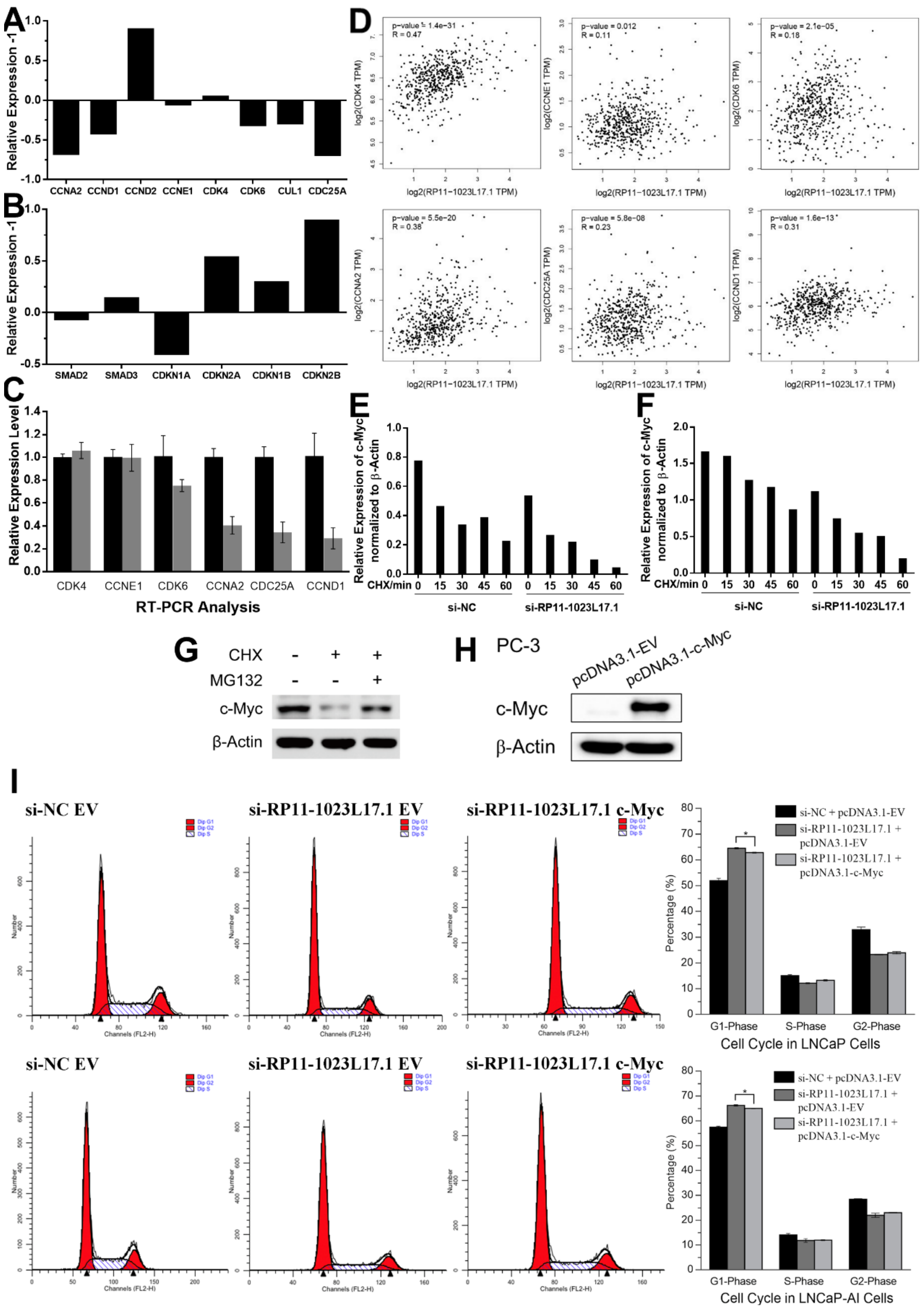

Supplementary Figure S6. The expression of c-Myc target genes in RP11-1023L17.1 knockdown microarray analysis. (**A-B**) The expression of genes up (A) or down (B)-regulated by c-Myc in microarray data. (**C**) The relative expression of c-Myc target genes in LNCaP cells transfected with si-NC or si-RP11-1023L17.1 were measured by qRT-PCR. Data are presented as mean ± SD (n ≥3). (**D**) Analysis of the expression correlation between RP11-1023L17.1 and c-Myc target genes in PCa and normal tissues of the TCGA database. (**E-F**) Quantification of c-Myc normalized on β-Actin in LNCaP(E) and 22Rv1(F) and represented in the bars graph (**G**) Assessment of c-Myc expression

by western blotting in LNCaP cells treated with CHX and/or MG132. (**H**) Assessment of c-Myc expression by western blotting in PC-3 cells transfected with pcDNA3.1-EV or pcDNA3.1-c-Myc. β-actin was used as a loading control. (**I**) The LNCaP and LNCaP-AI cells co-transfected with si-NC or si-RP11-1023L17.1 and EV or pcDNA3.1-c-Myc were stained with propidium iodide (PI), and the cell cycle distributions were evaluated by flow cytometry. *$p < 0.05$, and data are presented as mean ± SD ($n \geq 3$).

Table S1. Sequences of siRNAs

siRNA	Sequence
si-NC	5′- UUCUCCGAACGUGUCACGUTT - 3′
si- RP11-1023L17.1	5′- GGCCCGAUGGUCUUCAUAATT - 3′
si- c-Myc	5′- AGACCUUCAUCAAAAACAUTT - 3′
si-Dicer	5′- AAGGCUUACCUUCUCCAGGCT - 3′
si-AR-544	5′- GACAGUGUCACACAUUGAATT - 3′
si-AR-357	5′- GGAGCUCUCACAUGUGGAATT - 3′
si-FBXO32	5′- CUUGUCCGAUGUUACCCAATT - 3′

Table S2. Sequences of primers

Use	Primer	Sequence
qRT-PCR	RP11-1023L17.1-F	5′- GATTTACTTTTGGGATACACTCATCAT - 3′
qRT-PCR	RP11-1023L17.1-R	5′- ACAGGTGACTTCATCTTGGATTT - 3′
qRT-PCR	c-Myc -F	5′- GCGAACACACAACGTCTTGG - 3′
qRT-PCR	c-Myc -R	5′- CTACCTTGGGGGCCTTTTCA - 3′
qRT-PCR	β-actin-F	5′- CCTCTCCCAAGTCCACACAG - 3′
qRT-PCR	β-actin-R	5′- GGGCACGAAGGCTCATCATT - 3′
qRT-PCR	AR-F	5′- GCCTTGCTCTCTAGCCTCAA - 3′
qRT-PCR	AR-R	5′- GGTCGTCCACGTGTAAGTTG - 3′
qRT-PCR	KLK3-F	5′- GTGCTTGTGGCCTCTCGT - 3′
qRT-PCR	KLK3-R	5′- CAGCAAGATCACGCTTTTGT - 3′
CHIP-PCR	RP11-1023L17.1-ARE -F-1	5′- GGCAGTCTTGGGACTTGGG-3′
CHIP-PCR	RP11-1023L17.1-ARE -R-1	5′- AACAGGGGCTTCGGGTG-3′
CHIP-PCR	RP11-1023L17.1-ARE -F-2	5′- CATTGAGTAGTGACCCCGACG -3′
CHIP-PCR	RP11-1023L17.1-ARE -R-2	5′- GGATGCCAGCATGAGAAGAAC -3′
CHIP-PCR	KLK3-ARE -F	5′- TGGGACAACTTGCAAACCTG-3′
CHIP-PCR	KLK3-ARE -R	5′- CCAGAGTAGGTCTGTTTTCAATCCA-3′
CHIP-PCR	XBP-ARE -F	5′- TCTGGAAAGCTCTCGGTTTG 3′
CHIP-PCR	XBP-ARE -R	5′- AATCCCTGGCCAAAGGTACT 3′
Plasmid construction	c-Myc-clone-F	5′- CTGGATTTTTTTCGGGTAGTGG -3′
Plasmid construction	c-Myc-clone-R	5′- TTACGCACAAGAGTTCCGTAGC -3′
Plasmid construction	RP11-1023L17.1-T7-F	5′- TAATACGACTCACTATAGGGGGGAACAGAGCAACAGTCTC -3′
Plasmid construction	RP11-1023L17.1-T7-R	5′- ATTAAGCTAGGACGTGACTTGG -3′
